# Impact of serum bilirubin levels and preoperative biliary drainage on perioperative complications of pancreaticoduodenectomy

**DOI:** 10.3389/fmed.2025.1535106

**Published:** 2025-05-14

**Authors:** Yilan Li, Sicheng Pu, Kezhen Zong, Rui Liao, Zuotian Huang, Jianbo Huang, Yawen Liu, Baoyong Zhou, Zhongjun Wu

**Affiliations:** ^1^Department of Hepatobiliary Surgery, The First Affiliated Hospital of Chongqing Medical University, Chongqing, China; ^2^Department of Hepatobiliary Pancreatic Tumor Center, Chongqing University Cancer Hospital, Chongqing, China; ^3^Department of Breast and Thyroid Surgery, The First Affiliated Hospital of Chongqing Medical University, Chongqing, China; ^4^Department of Hepatobiliary Surgery, Bishan Hospital of Chongqing Medical University, Chongqing, China

**Keywords:** pancreaticoduodenectomy, preoperative serum bilirubin, preoperative biliary drainage, restricted cubic splines, complication

## Abstract

**Background:**

The effect of elevated preoperative serum bilirubin on complications after pancreaticoduodenectomy (PD) remains uncertain. Preoperative biliary drainage (PBD) effectively reduces serum bilirubin values; however, its impact on PD complications remains debatable.

**Methods:**

We conducted a retrospective analysis on patients who underwent PD at the First Affiliated Hospital of Chongqing Medical University from October 2018 to July 2023. Patients were categorized into quartiles based on preoperative serum bilirubin levels. Multivariable logistic regression was used to investigate the relationship between bilirubin and the risk of PD complications. Restricted cubic spline (RCS) analysis was conducted to assess the dose–response relationship between bilirubin levels and PD complications. Furthermore, a 3-way interaction of PD complications, preoperative serum bilirubin, and PBD was examined.

**Results:**

A total of 326 patients participated in the study. Multivariate logistic regression analysis revealed that higher preoperative serum bilirubin levels increased the likelihood of perioperative PD complications. RCS analysis revealed a significant linear dose–response relationship between bilirubin levels and the risk of PD complications. While PBD did not independently affect PD complications, there was a significant 3-way interaction of PD complications, preoperative serum bilirubin levels, and PBD, indicating that patients with serum bilirubin values exceeding 10 times the upper limit of normal (≥ 171 μmol/L) exhibited a lower risk of complications through PBD.

**Conclusion:**

Elevated preoperative serum bilirubin increases the risk of complications after PD. Patients with high preoperative serum bilirubin values (≥ 171 μmol/L) undergoing PD benefit from PBD.

## 1 Introduction

Pancreaticoduodenectomy (PD) is the classical surgical procedure for treating both benign and malignant diseases of the pancreas, duodenum, distal bile duct, and periampullary region. This complex and invasive operation is associated with a high incidence of complications (1). Studies have demonstrated that about 50% of patients undergoing PD experience various complications (2–4), significantly affecting the prognosis. Consequently, it is crucial to recognize patients with a higher risk of complications and allow for early intervention.

Malignant tumors of the peripancreatic area often cause mechanical obstruction of the bile duct, resulting in impaired bile drainage and significantly increased serum bilirubin levels (5). Some studies suggest that high levels of preoperative serum bilirubin are predictive of poor outcomes in patients with PD (6, 7), which can be used to identify high-risk patients. However, other perspectives argue that hyperbilirubinemia does not affect the morbidity and mortality of PD. The necessity of preoperative intervention remains debatable (8, 9). This study aimed to explore the link between preoperative serum bilirubin levels and PD complications, as well as the dose–response relationship between the two.

Preoperative biliary drainage (PBD) can effectively reduce preoperative serum bilirubin levels, mitigating adverse events such as nutritional deficiencies, infectious complications, acute renal failure, and coagulation disorders associated with hyperbilirubinemia (10–13). However, evidence suggests that PBD increases the risk of postoperative wound infection and intra-abdominal bleeding after PD (14, 15). Consequently, the relationship between preoperative serum bilirubin, PBD, and PD complications is still a matter of debate. This study aimed to address this issue and discuss the indications for PBD.

## 2 Materials and methods

### 2.1 Patient selection and data collection

This retrospective study included 326 patients who underwent PD at the First Affiliated Hospital of Chongqing Medical University from October 2018 to July 2023. Data collection comprised demographic information, laboratory data, surgical records, and postoperative outcomes. The exclusion criteria were as follows: severe cardiovascular and cerebrovascular diseases, hematological diseases, autoimmune diseases, PD combined with other organ resection, and minors. The study conformed to the standards outlined by the Declaration of Helsinki and received approval from the Ethics Committee of the First Affiliated Hospital of Chongqing Medical University (Approval no. K2023-322).

### 2.2 Surgical procedure and perioperative management

PD was performed by experienced surgeons using either open or laparoscopic methods. Digestive tract reconstruction was conducted using the classic Child’s method, with pancreatojejunostomy primarily performed via a duct-to-mucosa anastomosis. All patients received prophylactic antibiotic treatment during the perioperative period. The standard regimen included intravenous administration of cefoperazone-sulbactam (3 g/8 h) initiated 30 min before the skin incision and continued for 24 h postoperatively. Subsequent adjustments were made based on postoperative laboratory markers (e.g., leukocytosis, CRP elevation) and microbiological culture results. For patients with a history of penicillin allergy, levofloxacin (500 mg/24 h) was used as an alternative. Typically, octreotide or somatostatin therapy was administered for 5–7 days following the surgery. After the operation, patients continued to take acid suppressants for several months. Early in the postoperative period, patients received parenteral nutrition before gradually switching to enteral nutrition.

The timing of PBD was determined by the attending surgeon’s clinical judgment, prioritizing factors such as: severity of jaundice (bilirubin levels), nutritional status, comorbidities, and tumor resectability on imaging. Our institutional protocol generally aims for 7–14 days of PBD to allow bilirubin reduction and liver function recovery. The exact duration varied based on rate of bilirubin decline, resolution of cholangitis (if present), and patient’s physiological readiness for major surgery. The primary goal of PBD was not strict normalization but rather reduction to a safer threshold (usually <10 times the normal value in clinical applications). PBD techniques include percutaneous and endoscopic biliary drainage. The percutaneous routes consist of percutaneous transhepatic drainage, while endoscopic routes comprise both endoscopic nasobiliary drainage and endoscopic retrograde cholangiopancreatography with stent placement.

### 2.3 Definition and evaluation

The primary outcomes were defined as the occurrence of any PD complications, including clinical postoperative pancreatic fistula (POPF), intra-abdominal bleeding, intra-gastrointestinal bleeding, Clavien-Dindo grade III (CD3) complications (or higher than grade III), surgical site infection (SSI), postoperative transfusion, admission to the intensive care unit (ICU), reoperation, and readmission. Secondary outcomes included operative time, intraoperative blood loss, total hospital stay, and postoperative hospital stay. Clinical POPF was diagnosed and classified according to the International Study Group on Pancreatic Surgery criteria, which categorizes POPF into biochemical leak, Grade B, and Grade C (16). Clinical POPF incorporates both Grade B and Grade C POPF. Intra-abdominal bleeding was characterized by the presence of hemorrhagic fluid in the abdominal drain or non-cavitary bleeding associated with an unexplained decrease in hemoglobin levels, confirmed by imaging or endoscopy and requiring transfusion, endoscopic or surgical hemostasis. Intra-gastrointestinal bleeding was identified by melena, persistent black stool, or blood in the gastric tube drain, confirmed by imaging or endoscopy, and requiring appropriate hemostatic treatment. SSI was defined according to the Centers for Disease Control and Prevention guidelines (17). Readmission was defined as any hospital admission within 90 days postoperatively. Reoperation was referred to as any unplanned second operation during the hospital stay.

### 2.4 Statistical analysis

The study cohort was divided into four groups based on the 25th, 50th, and 75th percentiles of preoperative serum bilirubin levels. Baseline characteristics and clinical outcomes of patients were compared in each group. Continuous variables are expressed as medians with interquartile ranges or means with standard deviations, depending on their distribution, and compared using the Kruskal–Wallis rank sum test. Categorical variables are expressed as frequencies and percentages and compared using Pearson’s Chi-squared test or Fisher’s exact test.

Multivariable logistic regression analysis was conducted to analyze the independent association between preoperative serum bilirubin levels and various perioperative complications. Model 0 was unadjusted, while Model 1 was adjusted for age, gender, body mass index (BMI), history of abdominal surgery, and neoadjuvant therapy. Moreover, Model 2 was adjusted for albumin, alanine aminotransferase (ALT), aspartate aminotransferase pressure (AST), main pancreatic duct diameter (MPD), surgical approach, and pathological diagnosis.

A dose–response relationship between a continuous exposure and an outcome can be effectively described using restricted cubic spline functions, which can also be used to statistically and/or visually verify the association’s assumed linearity (18). A RCS model with three knots (at the 10th, 50th, and 90th percentiles) was utilized to explore the dose–response relationship between bilirubin levels and the risk of PD complications.

To evaluate if preoperative serum bilirubin and PBD have a significant effect on PD complications, we examined a 3-way interaction of PD complications × bilirubin (as a continuous variable) × PBD using an interactive restricted spline. First, the 2-way interaction of bilirubin and PBD was tested in the adjusted logistic model. If a significant interaction was observed, further analysis was performed to determine a potential bilirubin threshold. By comparing the outcomes of PBD and non-PBD based on this threshold, patients benefiting from either procedure through bilirubin may be identified. In our study, all statistical analyses were performed using R software (version 4.2.2), along with the use of MSTATA software. A *p* < 0.05 was considered statistically significant.

## 3 Results

### 3.1 Baseline characteristics of the participants

**Figure 1:**
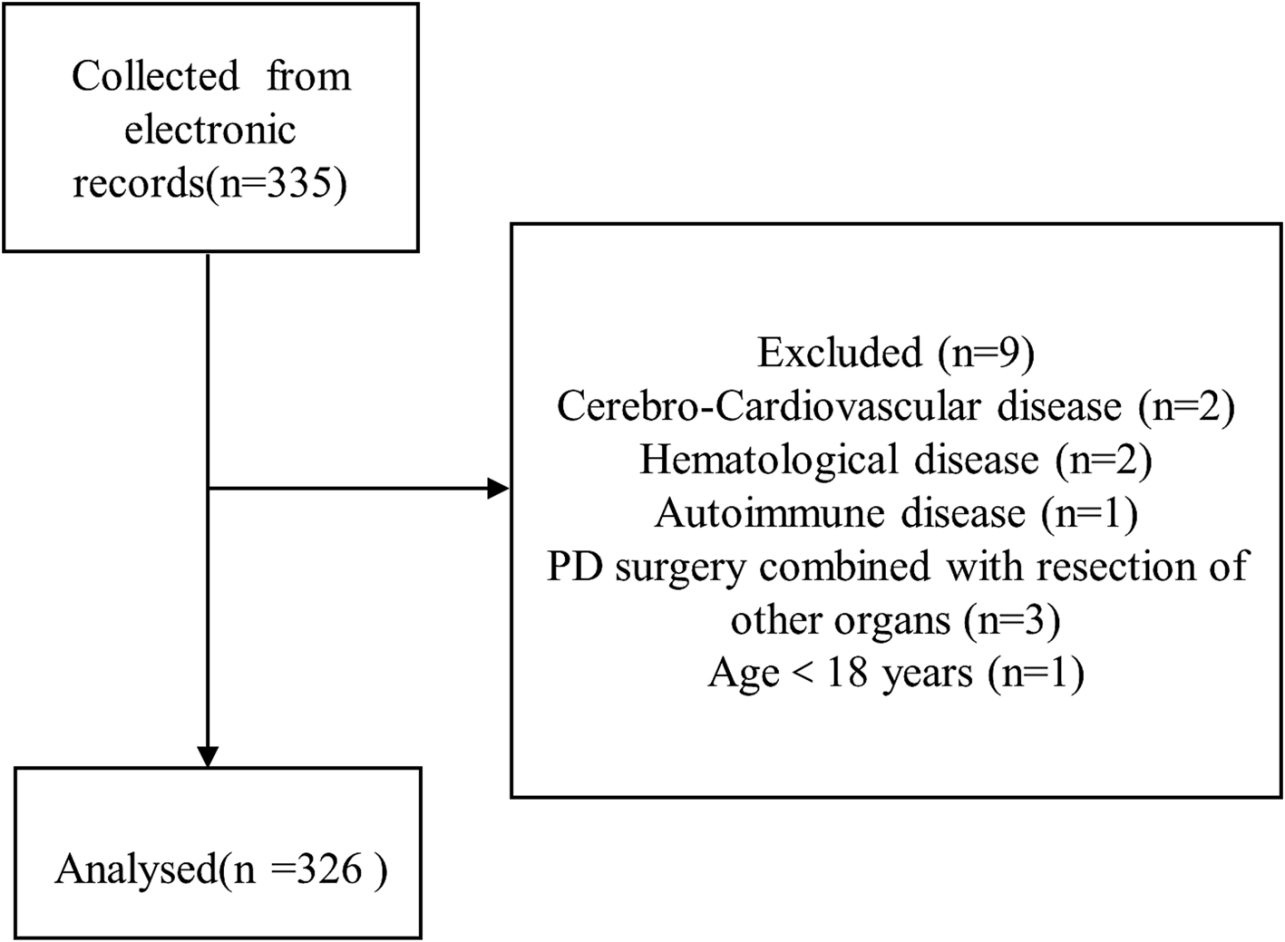
The flowchart of inclusion and exclusion of patients.

A total of 335 patients were initially included in the study. After excluding nine cases, 326 patients remained for analysis (Figure 1). Table 1 displays the patients’ baseline characteristics grouped by preoperative bilirubin quartiles. Age, gender, BMI, hypertension, diabetes, drinking, smoking, neoadjuvant chemotherapy, MPD, and history of abdominal surgery were statistically non-significant between the four groups. PBD, surgical approach, and pathologic diagnosis exhibited statistically significant differences (*p* < 0.05). The albumin, ALT, and AST also exhibited significant differences (*p* < 0.05), while the other laboratory makers were statistically non-significant.

**Table 1:** Baseline characteristics of participants based on quartiles of total bilirubin.

Characteristics	Q1	Q2	Q3	Q4	*P*-value
Age (years)	62 (51, 68)	64 (55, 67)	62 (56, 69)	63 (52, 68)	0.873
Gender					0.292
Male	45 (56%)	50 (60%)	56 (69%)	55 (67%)	
Female	35 (44%)	33 (40%)	25 (31%)	27 (33%)	
BMI (kg/m^2^)	21.6 (19.9, 24.0)	22.3 (19.7, 24.2)	22.5 (20.5, 24.8)	22.0 (20.1, 24.7)	0.523
Hypertension	18 (23%)	17 (20%)	13 (16%)	16 (20%)	0.774
Diabetes	13 (16%)	11 (13%)	15 (19%)	11 (13%)	0.755
Drink	24 (30%)	24 (29%)	22 (27%)	23 (28%)	0.982
Smoke	33 (41%)	31 (37%)	31 (38%)	35 (43%)	0.887
Neoadjuvant chemotherapy	2 (2.5%)	0 (0%)	0 (0%)	1 (1.2%)	0.151
PBD					<0.001
Percutaneous	0 (0.0%)	3 (3.6%)	23 (28.4%)	31 (37.8%)	
Endoscopic	5 (6.3%)	12 (14.5%)	16 (19.8%)	23 (28.0%)	
None	75 (93.8%)	68 (81.9%)	42 (51.9%)	28 (34.1%)	
MPD (mm)	3.70 (2.66, 5.89)	3.87 (2.88, 5.31)	3.81 (2.69, 5.97)	3.93 (2.66, 5.87)	0.994
History of abdominal surgery	22 (28%)	22 (27%)	13 (16%)	15 (18%)	0.195
White blood cell (10^9^/L)	5.55 (4.57, 6.93)	5.35 (4.53, 6.85)	5.49 (4.65, 6.54)	6.01 (5.16, 7.35)	0.106
Albumin (g/L)	41.5 (38.0, 44.0)	39.0 (35.3, 42.0)	38.0 (35.0, 42.0)	36.5 (34.0, 39.8)	<0.001
ALT (U/L)	23 (15, 41)	133 (61, 215)	228 (128, 370)	151 (89, 226)	<0.001
AST (U/L)	23 (18, 30)	88 (47, 169)	142 (82, 232)	104 (68, 190)	<0.001
Surgical approach					0.035
LPD	47 (59%)	56 (67%)	47 (58%)	34 (41%)	
OPD	26 (33%)	22 (27%)	27 (33%)	34 (41%)	
LPD convert to OPD	7 (8.8%)	5 (6.0%)	7 (8.6%)	14 (17%)	
Pathologic diagnosis					0.001
Bile duct cancer	5 (6.3%)	12 (14%)	20 (26%)	22 (28%)	
Pancreatic cancer	43 (54%)	39 (47%)	40 (51%)	40 (50%)	
Ampullary cancer	1 (1.3%)	9 (11%)	5 (6.4%)	8 (10%)	
Duodenal cancer	16 (20%)	14 (17%)	8 (10%)	7 (8.8%)	
IPMN	5 (6.3%)	2 (2.4%)	0 (0%)	0 (0%)	
Chronic pancreatitis	2 (2.5%)	3 (3.6%)	2 (2.6%)	1 (1.3%)	
Liver cancer	1 (1.3%)	0 (0%)	0 (0%)	0 (0%)	
Others	7 (8.8%)	4 (4.8%)	3 (3.8%)	2 (2.5%)	

Data are presented as median (interquartile range) or *n* (%). BMI, body mass index; PBD, preoperative biliary drainage; MPD, main pancreatic duct diameter; ALT, alanine aminotransferase; AST, aspartate aminotransferase pressure; LPD, laparoscopic pancreaticoduodenectomy; OPD, open pancreaticoduodenectomy; IPMN, intraductal papillary mucinous neoplasms.

### 3.2 Association between bilirubin and complications

**Figure 2:**
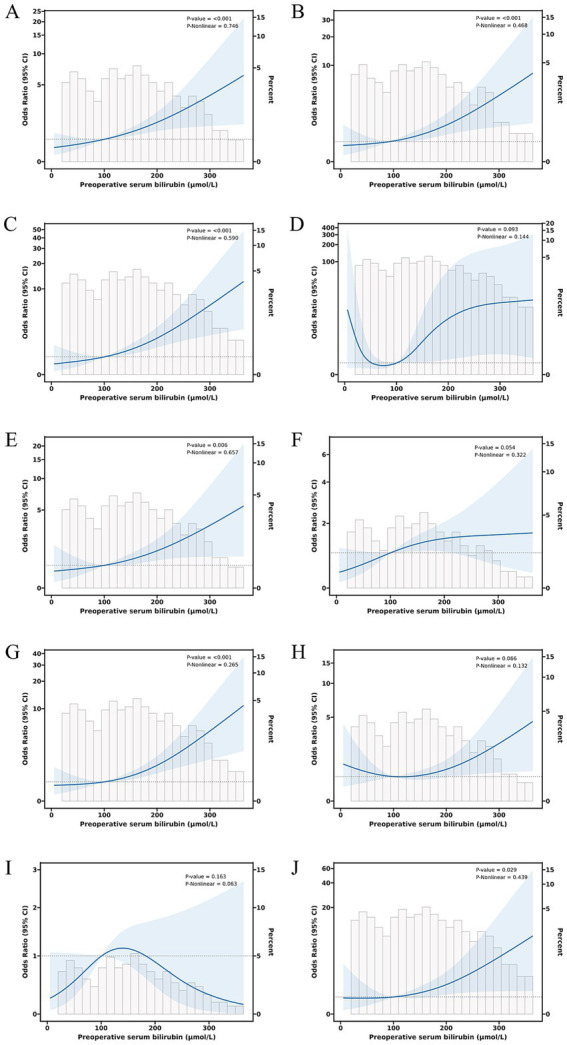
Dose–response relationship between preoperative serum bilirubin and risk of any complication **(A)**, clinical POPF **(B)**, intra-abdominal bleeding **(C)**, intra-gastrointestinal bleeding **(D)**, CD3 complications **(E)**, SSI **(F)**, postoperative transfusion **(G)**, ICU admission **(H)**, reoperation **(I)**, and readmission **(J)**.

**Figure 3:**
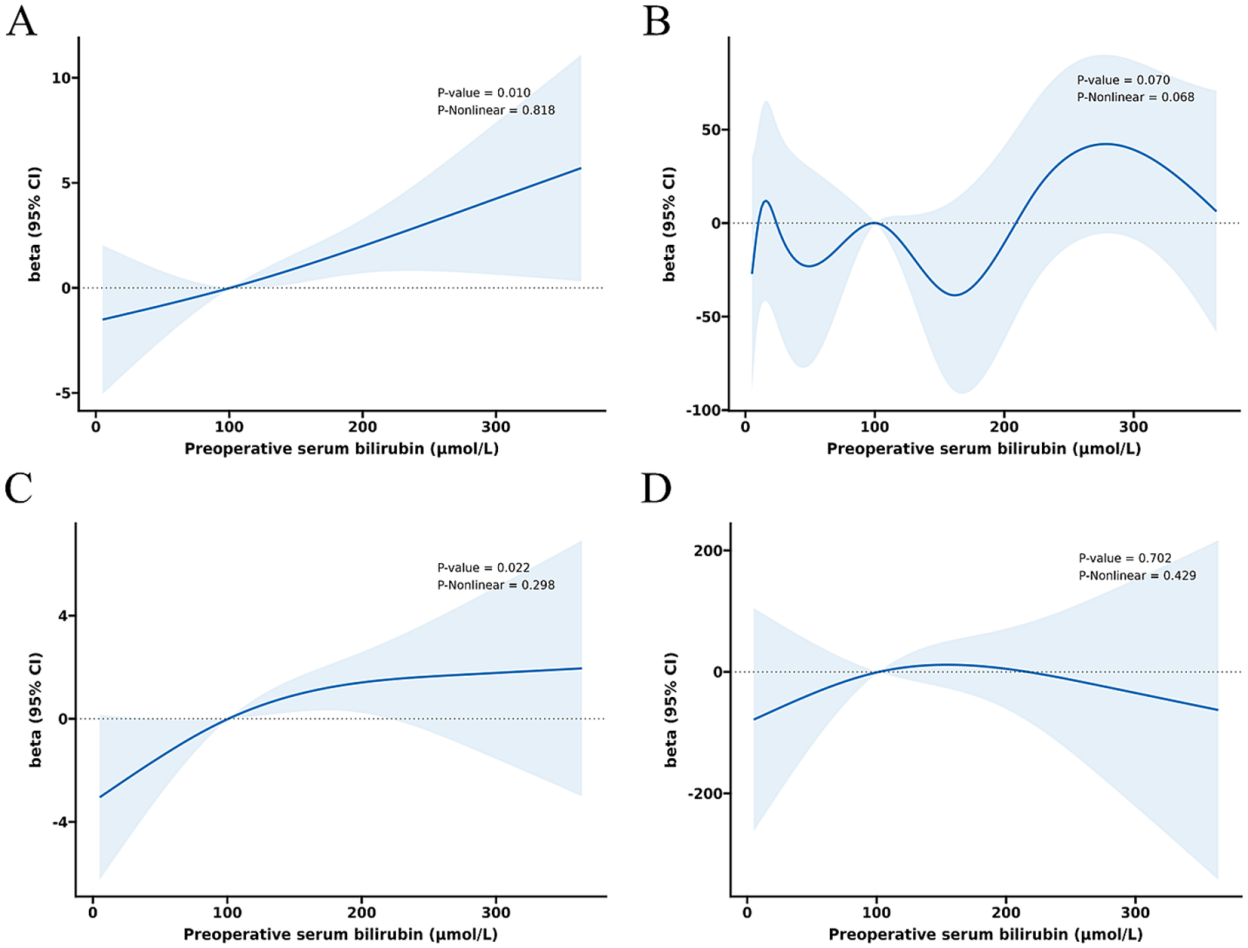
Dose–response relationship between preoperative serum bilirubin and risk of operative time **(A)**, intraoperative blood loss **(B)**, total hospital stay **(C)**, and postoperative hospital stay **(D)**.

The relationship between total serum bilirubin and perioperative PD complications is detailed in Tables 2, 3. Using the lowest quartile as the reference, the highest quartile of total bilirubin (TB) was significantly associated with any complication in all three logistic regression models. The odd ratios (OR) and 95% confidence intervals (CIs) were 2.60 (1.38 and 4.90), 2.67 (1.38 and 5.14), and 4.60 (1.90 and 11.1). Similarly with clinical POPF, OR and 95% CIs were 2.19 (1.09 and 4.38), 2.22 (1.09 and 4.54), and 4.21 (1.40 and 12.6). TB in the highest quartile of Models 0, 1, and 2 were associated with intra-abdominal bleeding compared to reference, with ORs and 95% CIs of 3.59 (1.43 and 9.01), 3.58 (1.41 and 9.14), and 12.8 (3.32 and 49.0). Additionally, the highest quartile of bilirubin was significantly associated with postoperative transfusion in Model 0 (OR: 2.56, 95% CI: 1.26 and 5.18), Model 1 (OR: 2.41, 95% CI: 1.18 and 4.92), and Model 2 (OR: 4.35, 95% CI: 1.59 and 11.9). Although the highest quartile of bilirubin was unrelated to readmission, the second quartile (OR: 4.38, 95% CI: 1.12 and 17.2) and third quartile (OR: 5.43, 95% CI: 1.01 and 29.2) exhibited significant associations in Model 2. Moreover, Table 2 indicates that there is an increased risk of any complication, clinical POPF, intra-abdominal bleeding, SSI, and postoperative transfusion with increasing TB (*p-*values for trend were 0.002, 0.013, 0.001, 0.044, and < 0.001, respectively). RCS analysis (Figures 2, 3) revealed a significant linear dose–response relationship between bilirubin levels and any complication, clinical POPF, intra-abdominal bleeding, CD3 complication, postoperative transfusion, reoperation, operative time, and total hospital stay (*P* for overall < 0.05, P for non-linearity > 0.05).

**Table 2:** OR (95% CI) for risk of complications according to quartiles of total bilirubin.

Variables	Model 0	Model 1	Model 2	*P*-value for trend
Any complication				0.002
Q1	Ref	Ref	Ref	
Q2	1.15 (0.62, 2.14)	1.11 (0.59, 2.11)	1.55 (0.71, 3.38)	
Q3	1.39 (0.75, 2.60)	1.36 (0.72, 2.60)	2.24 (0.91, 5.52)	
Q4	2.60 (1.38, 4.90)*	2.67 (1.38, 5.14)*	4.60 (1.90, 11.1)*	
Clinical POPF				0.013
Q1	Ref	Ref	Ref	
Q2	0.93 (0.45,1.94)	0.95 (0.45, 2.01)	1.04 (0.37, 2.91)	
Q3	0.95 (0.45, 1.98)	0.96 (0.45, 2.03)	1.52 (0.46, 4.99)	
Q4	2.19 (1.09, 4.38)*	2.22 (1.09, 4.54)*	4.21 (1.40, 12.6)*	
Intra-abdominal bleeding				0.001
Q1	Ref	Ref	Ref	
Q2	1.11 (0.38, 3.22)	1.06 (0.36, 3.10)	2.13 (0.58, 7.76)	
Q3	1.47 (0.53, 4.07)	1.40 (0.50, 3.94)	4.10 (0.98, 17.1)	
Q4	3.59 (1.43, 9.01)*	3.58 (1.41, 9.14)*	12.8 (3.32, 49.0)*	
Intra-gastrointestinal bleeding				0.134
Q1	Ref	Ref	Ref	
Q2	0.71 (0.15, 3.29)	0.68 (0.15, 3.20)	0.15 (0.01, 1.85)	
Q3	2.68 (0.80, 8.92)	2.36 (0.69, 8.09)	0.32 (0.03, 3.01)	
Q4	1.77 (0.50, 6.31)	1.59 (0.44, 5.79)	0.97 (0.13, 7.48)	
CD3 complication				0.052
Q1	Ref	Ref	Ref	
Q2	0.78 (0.32, 1.91)	0.76 (0.30, 1.92)	0.86 (0.26, 2.82)	
Q3	0.89 (0.37, 2.15)	0.72 (0.29, 1.80)	1.06 (0.29, 3.93)	
Q4	1.83 (0.83, 4.04)	1.64 (0.71, 3.76)	3.21 (0.98, 10.5)	
Surgical site infection				0.044
Q1	Ref	Ref	Ref	
Q2	0.57 (0.25, 1.30)	0.56 (0.24, 1.30)	0.89 (0.29, 2.74)	
Q3	0.98 (0.46, 2.10)	0.97 (0.45, 2.10)	2.24 (0.66, 7.59)	
Q4	1.53 (0.75, 3.14)	1.51 (0.73, 3.15)	2.77 (0.89, 8.60)	
Postoperative transfusion				<0.001
Q1	Ref	Ref	Ref	
Q2	0.81 (0.37, 1.80)	0.76 (0.34, 1.69)	1.01 (0.37, 2.79)	
Q3	1.68 (0.81, 3.48)	1.51 (0.72, 3.16)	1.78 (0.61, 5.18)	
Q4	2.56 (1.26, 5.18)*	2.41 (1.18, 4.92)*	4.35 (1.59, 11.9)*	
ICU admission				0.483
Q1	Ref	Ref	Ref	
Q2	0.69 (0.31, 1.53)	0.64 (0.28, 1.44)	0.71 (0.25, 2.05)	
Q3	0.46 (0.19, 1.11)	0.37 (0.15, 0.92)*	0.41(0.12, 1.43)	
Q4	1.20 (0.57, 2.49)	1.05 (0.49, 2.26)	1.41 (0.48, 4.13)	
Readmission				0.959
Q1	Ref	Ref	Ref	
Q2	1.82 (0.58, 5.70)	1.85 (0.59, 5.84)	4.38 (1.12, 17.2)*	
Q3	1.42 (0.43, 4.67)	1.53 (0.45, 5.12)	5.43 (1.01, 29.2)*	
Q4	1.40 (0.40, 4.61)	1.49 (0.45, 4.99)	2.32 (0.42, 12.9)	
Reoperation				0.518
Q1	Ref	Ref	Ref	
Q2	0.39 (0.10, 1.57)	0.37 (0.09, 1.48)	0.29 (0.05, 1.71)	
Q3	0.99 (0.33, 2.95)	0.89 (0.29, 2.72)	1.00 (0.19, 5.15)	
Q4	0.97 (0.33, 2.91)	0.90 (0.30, 2.75)	1.75 (0.36, 8.48)	

OR, hazard ratio; CI, confidence interval; POPF, Postoperative Pancreatic Fistula; CD3, Clavien-Dindo grade III; ICU, Intensive Care Unit. *p < 0.05. Model 0: unadjusted. Model 1: adjusted for Age, Gender, BMI, History of abdominal surgery and Neoadjuvant chemotherapy. Model 2: adjusted for Albumin, ALT, AST, MPD, surgical approach and pathologic diagnosis on the basis of Model 1.

**Table 3:** Difference in LS mean (95% CI) for risk of complications according to quartiles of total bilirubin.

Variables	Model 0	Model 1	Model 2	*P*-value for trend
Operative time				0.649
Q1	Ref	Ref	Ref	
Q2	20.45 (−56.31, 15.40)	21.81 (−13.80, 57.41)	21.47 (−16.91, 59.85)	
Q3	−4.10 (−40.14, 31.93)	- 5.46 (−41.61, 30.69)	−0.53 (−43.75, 42.70)	
Q4	19.54 (−16.39, 55.47)	17.49 (−18.28, 53.27)	35.02 (−6.46, 76.50)	
Intraoperative blood loss				0.356
Q1	Ref	Ref	Ref	
Q2	74.64 (−87.99, 237.28)	76.25 (−87.93, 240.43)	104.93 (−87.88, 297.74)	
Q3	34.08 (−129.05, 197.21)	39.32 (−126.87, 205.50)	35.71 (−180.44, 251.85)	
Q4	104.16 (−58.48, 266.79)	108.62 (−55.84, 273.08)	51.09 (−156.33, 258.51)	
Total hospital stay				<0.001
Q1	Ref	Ref	Ref	
Q2	−1.20 (−4.56, 2.17)	−1.26 (−4.65, 2.12)	−0.67 (−4.37, 3.03)	
Q3	1.84 (−1.54, 5.23)	1.68 (−1.76, 5.12)	1.90 (−2.26, 6.07)	
Q4	4.53 (1.15, 7.91)*	4.40 (1.00, 7.80)	5.02 (1.03, 9.02)*	
Postoperative hospital stay				0.682
Q1	Ref	Ref	Ref	
Q2	−0.95 (−4.08, 2.18)	−0.95 (−4.09, 2.20)	−0.46 (−3.92, 3.00)	
Q3	1.61 (−1.54, 4.76)	1.47 (−1.72, 4.67)	1.91 (−1.99, 5.81)	
Q4	3.49 (0.35, 6.63)	3.35 (0.19, 6.52)	4.01 (0.27, 7.75)	

LS Mean, Least Squares Means; CI, confidence interval. *p < 0.05. Model 0: unadjusted. Model 1: adjusted for Age, Gender, BMI, History of abdominal surgery and Adjuvant chemotherapy. Model 2: adjusted for Albumin, ALT, AST, MPD, surgical approach and pathologic diagnosis on the basis of Model 1.

### 3.3 3-way interaction among PD complications, bilirubin, and PBD

**Figure 4:**
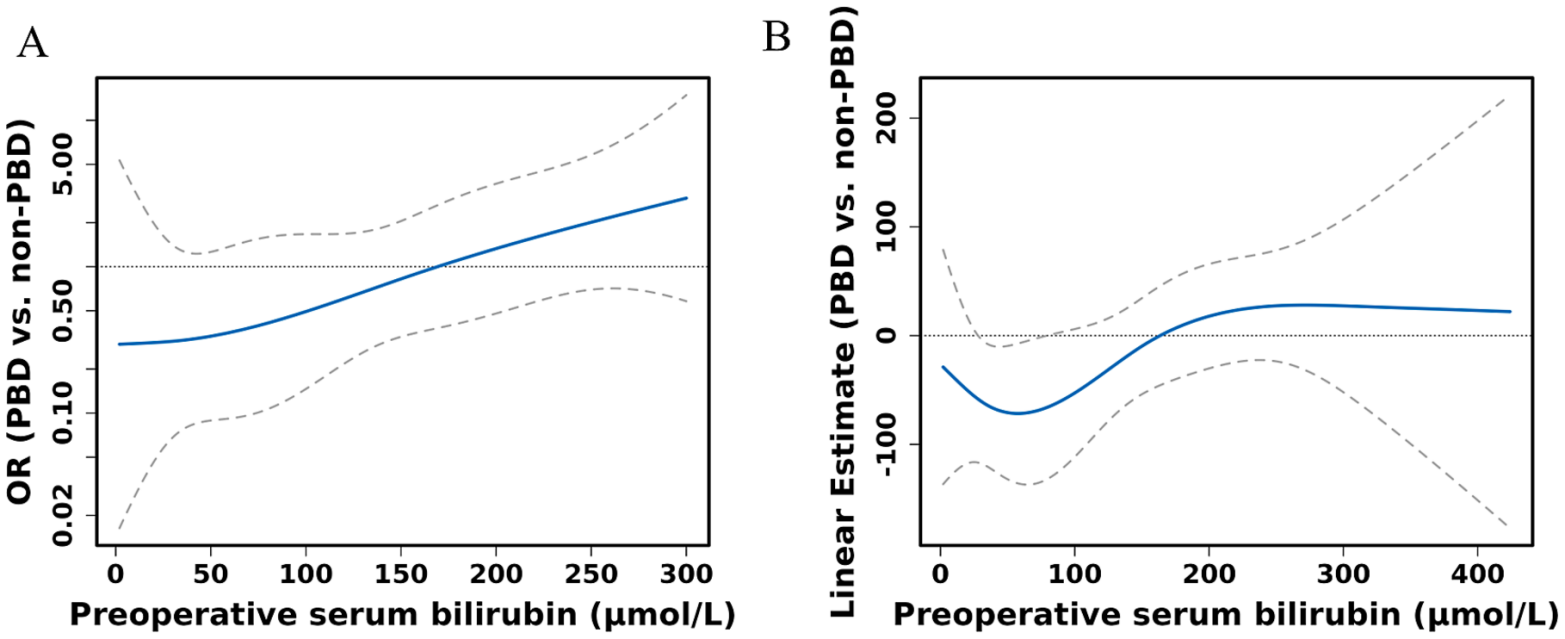
Spline figures plotting adjusted OR or linear estimate with PBD versus non-PBD for any complication **(A)** and operative time **(B)** based on preoperative serum bilirubin. Estimates derived from adjusted logistic models with RCS using knots at the 10th, 50th, and 90th percentiles.

**Figure 5:**
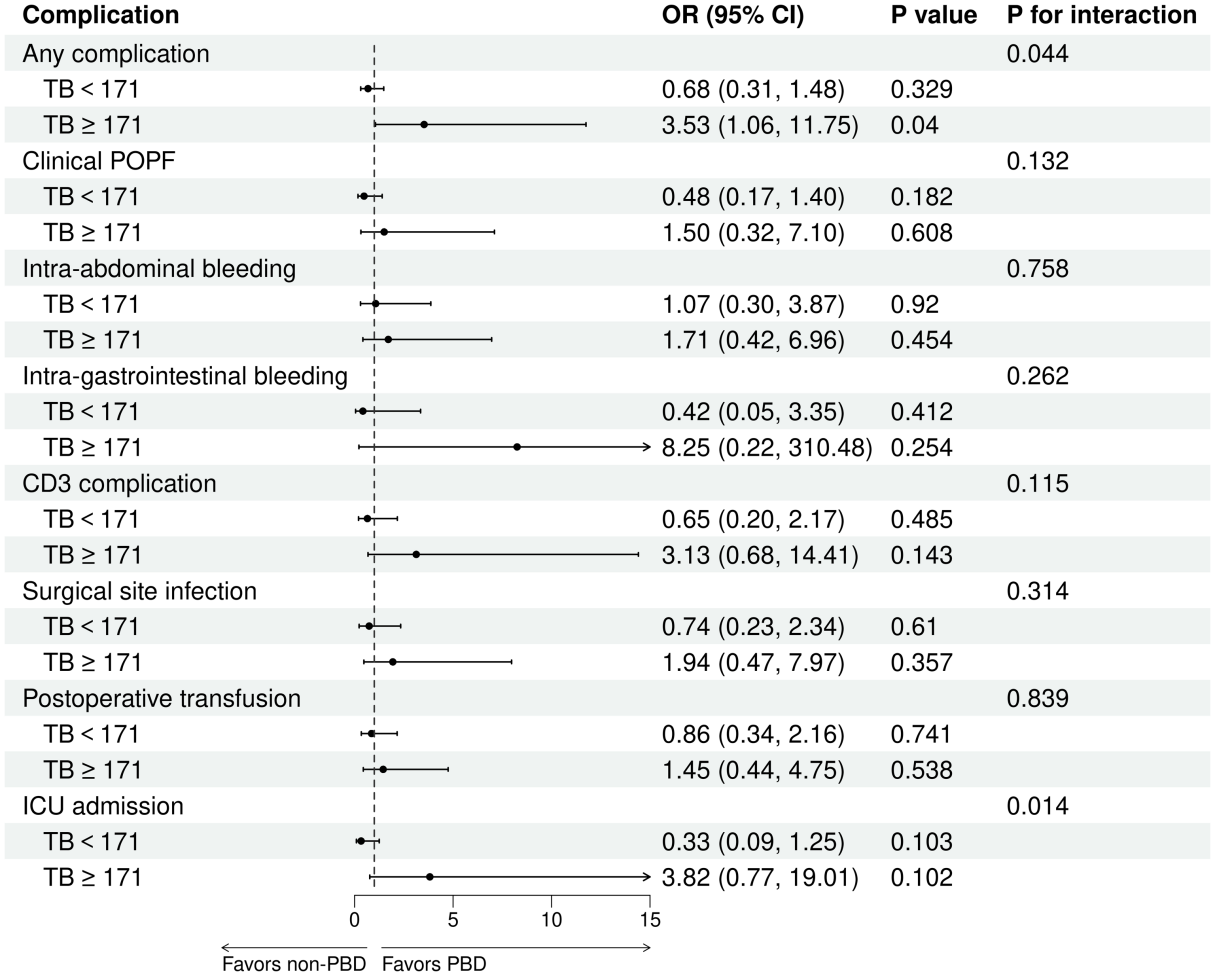
Patients with TB ≥ 171 μmol/L were associated with a decreased risk of any complication with PBD versus non-PBD. Forest plot estimates derived from adjusted logistic models for any complication, clinical POPF, intra-abdominal bleeding, intra-gastrointestinal bleeding, CD3 complications, SSI, postoperative transfusion, and ICU admission among patients undergoing PD.

**Figure 6:**
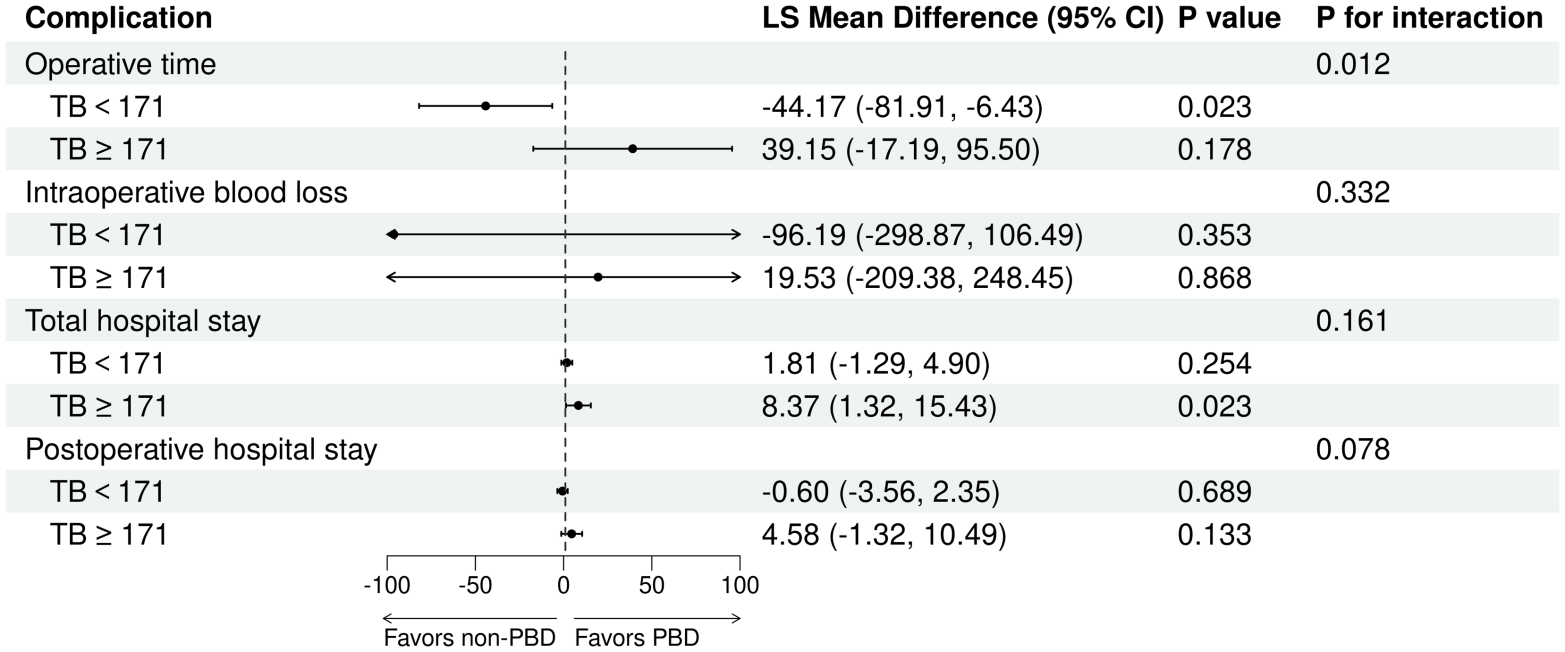
Patients with TB < 171 μmol/L were associated with increased operative time with PBD versus non-PBD. Forest plot estimates derived from linear models for operative time, intraoperative blood loss, total hospital stay, and postoperative hospital stay among patients undergoing PD.

The receipt of PBD was not independently associated with the risk of complications for patients undergoing PD (Supplementary Tables S1, S2). Subgroup analysis revealed that percutaneous biliary drainage was associated with a higher rate of Intra-gastrointestinal bleeding (15.8 vs. 3.6% in endoscopic, *p* = 0.028, Supplementary Table S3). However, a significant interaction was observed between bilirubin levels and PBD among PD patients (P for bilirubin × PBD interaction = 0.044). This suggests that patients with higher bilirubin exhibited a lower risk of any complication with PBD compared with non-PBD. Spline plots generated from the adjusted logistic model presented that OR for any complication following PD with PBD versus non-PBD varied with bilirubin values. The plots showed a threshold value of around 171 μmol/L, indicating that patients with bilirubin values ≥ 171 μmol/L benefited from PBD compared with non-PBD (OR for bilirubin ≥ 171 μmol/L with PBD versus non-PBD: 3.53; 95% CI: 1.06 and 11.75; *p* = 0.04). However, patients with bilirubin values < 171 μmol/L exhibited a similar risk of any complication with PBD and non-PBD (OR for bilirubin ≥ 171 μmol/L with PBD versus non-PBD: 0.68; 95% CI: 0.31 and 1.48; *p* = 0.329) (Figures 4A, 5). Similar results were not observed for other outcomes except operative time (P for bilirubin × PBD interaction = 0.012). Patients with bilirubin values < 171 μmol/L significantly declined in operative time without PBD compared with PBD (Least squares [LS] Mean Difference for bilirubin values < 171 μmol/L with PBD versus non-PBD: –44.17; 95% CI: −81.91 and −6.43; *p* = 0.023). Patients with bilirubin values ≥ 171 μmol/L exhibited similar operative time with PBD and non-PBD (LS Mean Difference for bilirubin ≥ 171 μmol/L with PBD versus non-PBD: 39.15; 95% CI: −17.19 and 95.50; *p* = 0.178) (Figures 4B, 6). Moreover, endoscopic biliary drainage is more frequent in patients with TB < 171 μmol/L (Supplementary Table S4).

## 4 Discussion

In this study, we analyzed the impact of preoperative serum bilirubin on perioperative complications of PD. Whether considered a categorical or continuous variable, bilirubin was associated with clinical POPF, intra-abdominal bleeding, postoperative transfusion, and length of hospital stay. RCS analysis indicated a linear relationship between bilirubin levels and these outcomes. Performing PBD in patients with TB more than 10 times the normal high value (TB ≥ 171 μmol/L) decreased the risk of any PD complication, while in patients with TB less than 10 times the normal high value (TB < 171 μmol/L), the operative time was prolonged undergoing PBD.

Mildly elevated serum bilirubin levels may offer health benefits, according to recent research (19, 20), which caused controversies on the prognosis of PD patients with high bilirubin values. Numerous studies support the notion that elevated serum bilirubin is detrimental. For instance, Wang et al. (21) identified preoperative bilirubin levels > 14.6 mg/dL (250 μmol/L) as an independent predictor of one-year mortality. Shen et al. (22) identified TB > 150 μmol/L as an independent poor prognostic factor. De Pastena et al. (23) found that bilirubin levels ≥ 7.5 mg/dL (128 μmol/L) significantly increased overall morbidity, acute kidney failure, reoperation rates, and length of hospital stay (23). Sauvanet et al. (6) multicenter study confirmed that severe hyperbilirubinemia (≥ 300 μmol/L) increased severe morbidity and decreased long-term survival in patients with pancreatic ductal adenocarcinoma undergoing PD (6). However, studies by van Gils et al. (9) and Scott Dolejs et al. (8) found no difference in postoperative complication rates between patients with bilirubin levels ≥ 250 μmol/L and those with lower levels (8, 9). Despite these differing opinions, our retrospective data indicated that increased bilirubin levels are disadvantageous due to higher rates of postoperative complications. Liver dysfunction, bleeding abnormalities, and malnutrition are common in cases of malignant obstructive jaundice (24). Moreover, the elevated risk of intra-abdominal bleeding and postoperative transfusion may be due to malignant tumors of the peripancreatic area that block the bile ducts, impairing vitamin K absorption and causing deficiency (25, 26). Vitamin K deficiency leads to an increased risk of bleeding and clotting disorders (27), as reported by Chen et al. (28).

Appropriate preoperative evaluation is crucial for favorable outcomes in patients with jaundice (10). There is no consensus on the cutoff point for elevated serum bilirubin that necessitates preoperative intervention. Receiver operating characteristic curves and generalized additive models were frequently utilized in previous research to determine threshold values for increased complication risks due to bilirubin (7, 23, 28). However, we utilized RCS analysis in our study, a methodology that was initially demonstrated in related fields. Although bilirubin was associated with several perioperative complications, all dose–response relationships were linear, making it difficult to determine an accurate cutoff value.

The value of PBD has been debated for years. PBD may benefit patients with jaundice by restoring intestinal function, improving liver function, and correcting coagulation abnormalities through bile drainage (10, 21, 29). Pattarapuntakul et al. (30) confirmed these benefits by comparing lower intraoperative bleeding and bile leakage rates to direct surgery, although without survival benefits. Conversely, van der Gaag et al.’s (31) multicenter randomized trial found that routine PBD increased severe complications in patients undergoing surgery for pancreatic head cancer (31). Ozgun et al. (14) reported higher overall complication rates in the PBD group compared to controls. They observed significant associations with wound infection, delayed gastric emptying, and intra-abdominal bleeding but not with postoperative pancreatic fistulas (14). El Nakeeb et al. (15) observed higher rates of major postoperative complications (pancreatic fistula, delayed gastric emptying, bile leakage, intra-abdominal bleeding, and wound infection) associated with PBD (32). While PBD effectively ameliorates hyperbilirubinemia, it may increase perioperative risks, potentially due to stent-induced bacterial colonization, pancreatitis, and cholangitis (31–33). A study by Mosquera et al. (34) stratified patients into three groups based on bilirubin values, revealing higher complication rates in those undergoing bile decompression compared to direct surgery when bilirubin levels were ≤ 10 mg/dL. Patients with bilirubin levels ≥ 15 mg/dL exhibited fewer complications and lower readmission rates, and no significant differences were observed when bilirubin levels ranged between 10.1 and 14.9 mg/dL. This indicated that the benefits and risks of PBD are determined by bilirubin values, as corroborated by our study. We found that performing PBD in patients with bilirubin above the cutoff value lowers the risk of complications while prolonging operative time with bilirubin below the cutoff value. This might be due to reduced bilirubin levels by PBD, although this invasive technique can lead to inflammation and fibrosis of the bile duct. Endoscopic stenting is particularly associated with these changes compared to percutaneous drainage, according to the prospective cohort study conducted by El-Haddad et al. (35). Numerous studies have also supported this finding (36, 37). This may explain why using PBD in patients with PD with slightly elevated bilirubin levels increases the risk of perioperative complications. However, our study only confirmed an extension in operative time, which may be due to the limited sample size. In conclusion, these findings provide surgeons with valuable insight when deciding whether to perform PBD before PD in clinical practice. Based on our study, it is recommended for patients with >10-fold normal bilirubin in our study. However, further large-scale studies are needed to confirm these results.

This study has several limitations. First, since it was a retrospective study conducted at a single institution, there could be issues with data loss and a limited sample size. Our findings may not generalize to other institutions with different surgical protocols or expertise levels. Second, we neglected to assess the effects of various PBD methods (percutaneous versus endoscopic) and stent types (metal versus plastic) on the results. Third, our retrospective analysis did not systematically collect ASA scores or detailed nutritional parameters, which limits our ability to fully adjust for these confounders in multivariable models. Besides, the impact of biliary drainage duration on complications remains unexplored in our cohort. Future studies should prospectively evaluate the optimal PBD-to-PD interval to balance jaundice resolution against procedural risks. Collaborative efforts should standardize data collection on drainage methods, stent types, and complication profiles to enable robust cross-institutional comparisons. These issues should be addressed in subsequent studies.

## 5 Conclusion

In general, preoperative elevation of bilirubin increases the risk of PD complications. PBD may reduce the overall rate of postoperative complications in patients with bilirubin levels more than 10 times the upper limit of normal. However, it could prolong operative time in patients with lower bilirubin levels.

## Data Availability

The original contributions presented in the study are included in the article/Supplementary material, further inquiries can be directed to the corresponding authors.
