# Supplementary material for: Impact of serum bilirubin levels and preoperative biliary drainage on perioperative complications of pancreaticoduodenectomy
Source: Front Med (Lausanne). 2025 May 14;12:1535106. doi: 10.3389/fmed.2025.1535106 (PMC12116578; doi:10.3389/fmed.2025.1535106)
Supplement: Supplementary file 1 [file Table_1.docx]

Table S1 Baseline characteristics of patients based on PBD status

| Variable | non-PBD  (N = 213) | PBD  (N = 113) | P Value |
| --- | --- | --- | --- |
| Age (years) | 63 (54, 68) | 62 (53, 68) | 0.672 |
| Gender |  |  | 0.021 |
| male | 125 (58.7%) | 81 (71.7%) |  |
| female | 88 (41.3%) | 32 (28.3%) |  |
| BMI (kg/m²) | 22.0 (20.3, 24.1) | 22.0 (20.1, 24.8) | 0.603 |
| Hypertension | 40 (18.8%) | 24 (21.2%) | 0.595 |
| Diabetes | 29 (13.6%) | 21 (18.6%) | 0.236 |
| Drink | 62 (29.1%) | 31 (27.4%) | 0.750 |
| Smoke | 82 (38.5%) | 48 (42.5%) | 0.485 |
| Neoadjuvant chemotherapy | 2 (0.9%) | 1 (0.9%) | >0.999 |
| MPD(mm) | 4.05 (2.69, 5.86) | 3.72 (2.67, 5.61) | 0.579 |
| History of abdominal surgery | 53 (24.9%) | 19 (16.8%) | 0.095 |
| Total serum bilirubin (μmol/L) | 32 (11, 128) | 182 (123, 259) | <0.001 |
| White blood cell (10^9^/L) | 5.33 (4.49, 6.60) | 6.14 (5.28, 7.59) | <0.001 |
| Albumin (g/L) | 40.0 (36.0, 43.0) | 37.0 (34.0, 41.0) | 0.001 |
| ALT(U/L) | 90 (25, 215) | 154 (76, 236) | <0.001 |
| AST(U/L) | 63 (23, 152) | 101 (66, 200) | <0.001 |
| Surgical approach |  |  | 0.432 |
| LPD | 125 (58.7%) | 59 (52.2%) |  |
| OPD | 66 (31.0%) | 43 (38.1%) |  |
| LPD convert to OPD | 22 (10.3%) | 11 (9.7%) |  |
| Pathologic diagnosis |  |  | 0.007 |
| Bile duct cancer | 28 (13.1%) | 31 (28.7%) |  |
| Pancreatic cancer | 116 (54.5%) | 46 (42.6%) |  |
| Ampullary cancer | 13 (6.1%) | 10 (9.3%) |  |
| Duodenal cancer | 32 (15.0%) | 13 (12.0%) |  |
| IPMN | 7 (3.3%) | 0 (0.0%) |  |
| Chronic pancreatitis | 6 (2.8%) | 2 (1.9%) |  |
| Liver cancer | 1 (0.5%) | 0 (0.0%) |  |
| Others | 10 (4.7%) | 6 (5.6%) |  |

Data are presented as median (interquartile range) or n (%). PBD, preoperative biliary drainage; BMI, body mass index; MPD, main pancreatic duct diameter; ALT, alanine aminotransferase; AST, aspartate aminotransferase pressure; LPD, laparoscopic pancreaticoduodenectomy; OPD, open pancreaticoduodenectomy; IPMN, intraductal papillary mucinous neoplasms.

Table S2  Perioperative outcomes and surgical-related parameters according to PBD status

| Variable | non-PBD  (N = 213) | PBD  (N = 113) | P Value |  |
| --- | --- | --- | --- | --- |
| Any complication | 100 (46.9%) | 59 (52.2%) | 0.366 |  |
| Clinical POPF | 52 (25.4%) | 33 (30.6%) | 0.326 |  |
| Intra-abdominal bleeding | 25 (12%) | 21 (19%) | 0.091 |  |
| Intra-gastrointestinal bleeding | 13 (6.1%) | 11 (9.7%) | 0.232 |  |
| CD3 complication | 31 (15%) | 22 (19%) | 0.252 |  |
| Surgical site infection | 40 (19%) | 29 (26%) | 0.148 |  |
| Postoperative transfusion | 50 (23%) | 36 (32%) | 0.102 |  |
| ICU admission | 38 (18%) | 21 (19%) | 0.868 |  |
| Readmission | 18 (8.5%) | 10 (8.8%) | 0.903 |  |
| Reoperation | 12 (5.6%) | 12 (11%) | 0.101 |  |
| Operative time | 410 (350, 480) | 400 (345, 465) | 0.391 |  |
| Intraoperative blood loss | 200 (200, 400) | 300 (200, 400) | 0.110 |  |
| Total hospital stay | 25 (20, 30) | 27 (24, 34) | <0.001 |  |
| Postoperative hospital stay | 16 (14, 21) | 17 (14, 21) | 0.319 |  |
| PBD, preoperative biliary drainage; POPF, postoperative pancreatic fistula; CD3, Clavien-Dindo grade III; ICU, intensive care unit. Covariates in the model included gender, bilirubin, white blood cell, albumin, ALT, AST, and pathologic diagnosis. | | | | |

Table S3 Perioperative outcomes and surgical-related parameters according to PBD methods

| Variable | Percutaneous (N = 57) Endoscopic ( N = 56) | | P Value |
| --- | --- | --- | --- |
| Any complication | 31 (54.4%) | 28 (50.0%) | 0.641 |
| Clinical POPF | 17 (30.9%) | 16 (30.2%) | 0.935 |
| Intra-abdominal bleeding | 10 (17.5%) | 11 (19.6%) | 0.774 |
| Intra-gastrointestinal bleeding | 9 (15.8%) | 2 (3.6%) | 0.028 |
| CD3 complication | 11 (19.3%) | 11 (19.6%) | 0.963 |
| Surgical site infection | 11 (19.3%) | 18 (32.1%) | 0.118 |
| Postoperative transfusion | 19 (33.3%) | 17 (30.4%) | 0.734 |
| ICU admission | 14 (24.6%) | 7 (12.5%) | 0.099 |
| Readmission | 6 (10.5%) | 4 (7.1%) | 0.742 |
| Reoperation | 6 (10.5%) | 6 (10.7%) | 0.974 |
| Operative time | 416 (345, 485) | 385 (331, 460) | 0.285 |
| Intraoperative blood loss | 300 (200, 400) | 300 (200, 400) | 0.642 |
| Total hospital stay | 27(23, 35) | 27(25, 33) | 0.685 |
| Postoperative hospital stay | 17 (14, 24) | 18 (14, 20) | 0.825 |
| PBD, preoperative biliary drainage; POPF, postoperative pancreatic fistula; CD3, Clavien-Dindo grade III; ICU, intensive care unit. Covariates in the model included gender, bilirubin, white blood cell, albumin, ALT, AST, and pathologic diagnosis. | | | |

Table S4 Comparison of PBD methods in patients with different total bilirubin levels

| Type of PBD | TB＜171 µmol/L  (N = 49) | TB ≥171 µmol/L  (N = 64) | P Value |  |
| --- | --- | --- | --- | --- |
| Percutaneous | 19 (38.8%) | 38 (59.4%) | 0.030 |  |
| Endoscopic | 30 (61.2%) | 26 (40.6%) |  |  |
| PBD, preoperative biliary drainage; TB, total bilirubin. | | | | |
